# Bilateral Recurrent Laryngeal Nerve Palsy following Total Thyroidectomy in Triple A Syndrome, an Unexpected but Critical Complication

**DOI:** 10.1155/2021/1315117

**Published:** 2021-11-19

**Authors:** Mathieu Chamberland, Marc-Antoine Poulin, Danielle Beaudoin

**Affiliations:** Hôpital de l'Enfant-Jésus, Québec (QC) G1J 1Z4 1401, 18 rue, Canada

## Abstract

**Introduction:**

Triple “A” syndrome (TAS) is a rare autosomal recessive disorder that presents in childhood with achalasia cardia, alacrima, ACTH-resistant adrenal insufficiency, with sensorimotor and autonomic polyneuropathy developing later in the course of the disease. *Case Presentation*. An adult white male affected by this syndrome underwent an uneventful total thyroidectomy for malignancy and suffered delayed bilateral recurrent laryngeal nerve palsy in the early postoperative hours. The palsy spontaneously resolved after a five-week course.

**Conclusion:**

Given the rarity of this severe condition and the absence of surgical or medical causes identifiable, there is possibility that it is the neurological involvement caused by TAS that predisposed the patient to this adverse outcome, precipitated by standard manipulations during surgery.

## 1. Introduction

Triple “A” syndrome (TAS) or Allgrove syndrome is a rare autosomal recessive disorder first described in 1978 [1]. Clinical manifestations encompass a classical triad of alacrima, achalasia cardia, and ACTH-resistant adrenal insufficiency, in addition with progressive sensorimotor and autonomic neuropathy. The disorder is caused by mutations in the AAAS gene on 12q13 chromosome coding for the nuclear pore protein ALADIN [2, 3].

Bilateral recurrent laryngeal nerve palsy (BRLNP) is a feared albeit rare complication of thyroid surgery and cervical central node dissection, arising in 0.4% of thyroid interventions [4]. The neuropathy can be permanent or transient. Symptom spectrum is influenced by the laterality of the lesions in addition to the position in which lies the vocal fold and ranges from voice hoarseness to airway compromise requiring surgical management with tracheostomy [2].

Cranial nerve involvement has been documented in the TAS neurological involvement continuum [2]. However, no report in literature suggests possible recurrent laryngeal dysfunction in this particular population, even less so in a postoperative setting.

## 2. Case Report

We present the case of a 32-year-old Caucasian male, referred to the department of otolaryngology for possibility of surgical management of a thyroid nodule demonstrating features of papillary thyroid carcinoma on a fine needle aspiration specimen.

The subject's medical history was remarkable for Allgrove syndrome, presenting at 7 years old with persistent esophageal dysphagia caused by achalasia cardia, treated with multiple pneumatic dilations in childhood and Heller cardiomyotomy with Toupet fundoplication at age 21. Cricopharyngeal myotomy was performed at age 26 to alleviate persistent symptoms oropharyngeal dysphagia. The patient's electronic records state normal vocal fold function assessed by flexible nasolaryngoscopy both pre and postoperatively at the time.

Neurological investigation had been undertaken at age 13 due to concerns regarding chronic hypertonus and gait disturbances. Evaluation confirmed spasticity in all four limbs and progressive motor axonal polyneuropathy on nerve conduction studies. Cerebral palsy was ruled out by the consulting specialist due to the progressive nature of the symptoms. Gait evaluation revealed no anomaly aside from tone and postural hypotension. The association of those two symptoms raised suspicion for the diagnosis of Allgrove syndrome, which was established on a clinical basis after documentation of ACTH-resistant hypoaldosteronism. On ophthalmology referral at time of diagnosis, Schirmer's test proved normal, indicating absence of alacrima. No specific genetic testing for Allgrove's syndrome was performed, as they were not available at time of diagnosis [5].

The subject's habitus was remarkable for significant chronic alcohol consumption and for occasional intranasal use of cocaine.

The patient was referred for opinion regarding surgical management of a papillary thyroid carcinoma. Decision was made to address the lesion with total thyroidectomy based on patient preference. Intervention was performed under vocal fold EMG monitoring with an endotracheal tube to provide continuous intraoperative neurophysiological monitoring (IONM). Direct laryngoscopy using conventional Macintosh #3 blade displayed a Cormack-Lehane grade 1 anatomy, and intubation was successfully performed. Visual assessment of adequate electrode placement at the vocal folds level with a second direct laryngoscopy was performed before final tube fixation. The surgery was well tolerated. Both RLNs were routinely dissected and preserved. Visual assessment confirmed bilateral nerve integrity, and neurostimulation with a 1 mA probing after procedure elicited a positive EMG vocal fold response of 600 mV and 1200 mV on the right and left sides, respectively. The patient was successfully extubated in the recovery room.

Inspiratory stridor developed within a few hours of the operation. Immediate evaluation ruled out local complications at the site of surgery, and fiberoptic nasolaryngoscopy demonstrated presence of movement of both vocal folds, but with limited fold abduction resulting in a noncritical diminished glottic aperture. Early expectant management was preferred, as patient's airway remained patent with vitals unchanged. Symptoms worsened over the first 48 h postoperative and culminated in airway compromise and respiratory distress requiring urgent intubation. The subject benefited from a surgical tracheotomy with a cuffed 7 mm tube 48 h postoperative to secure airway. He was progressed to a cuff-less tube as per hospital protocol and tolerated permanent obturation of the tracheotomy. The patient was discharged on postoperative day 11 with home tracheotomy care. Patient was reexamined under fiberoptic nasolaryngoscopy on postoperative day 14 and demonstrated signs of vocal fold movement recuperation. Laryngeal improvement remained stable on the 4th week follow-up. Upon demonstration of complete vocal fold movement recuperation on 5th week follow-up, tracheostomy was successfully weaned.

## 3. Discussion

TAS is a rare autosomal recessive condition, with at least 141 cases reported in literature [6]. It is caused by mutations in the AAAS gene, of which 65 mutations have been identified, although further genetic heterogeneity outside the AAAS gene is suspected [2, 6]. This syndrome usually presents in the first two decades of life by either achalasia cardia or adrenal insufficiency and the latter presenting mostly by glucocorticoid deficiency rather than mineralocorticoid deficiency. Alacrima is recognized as both the most consistent and earliest feature, but is commonly diagnosed retrospectively. Neurological features such as sensorimotor, autonomous, cerebellar, and cognitive impairments are common [7]. Additional features include cutaneous changes and dysmorphic features [7]. The proposed pathogenesis of this syndrome is the increased sensitivity to oxidative stress in specific tissue resulting from the mutation in the nuclear core protein ALADIN encoded by the AAS gene [8].

BRLNP is a rare but significant complication in the setting of anterior compartment neck surgery, as it can become rapidly life threatening and may require further surgical management to secure the airway. RLN injury has been reported to account for 22.2% of thyroid surgery complications and to occur in 4.3% of total thyroidectomies, with incidence of bilateral lesions estimated at 0.6% [4]. Most cases are caused by mechanical damage to RLNs, ranging from neurapraxia caused by stretching of the nerve during manipulation to axonotmesis or neurotmesis due to partial or complete transection. Postoperative mechanical damage mechanisms include hematoma formation and compression by scar tissue. Other mechanisms of injury include ischemic damage induced by inflammation [4].

Clinically significant palsies arise more frequently in the setting of partial or complete surgical transection of the nerve [4]. Respiratory distress therefore presents in the immediate postoperative period.

The subject presented with early progressive BLRNP following an uneventful total thyroidectomy for PTC. Anatomical integrity of the distal motor pathway was visually assessed and functionally demonstrated with a positive vocal fold EMG response elicited by 1 mA bilateral probing of RLN after dissection of the central compartment. The medical history and habitus also failed to provide a readily available explanation for this rare and dreaded surgical adverse event.

The patient was known for Allgrove syndrome with documented peripheral axonal polyneuropathic involvement. He suffered both motor and autonomous impairments, yet his medical records did not highlight CN dysfunction. CN involvement in the neuropathic spectrum of Allgrove's syndrome has been reported, mostly under the form of optic atrophy, lower CN weakness, and pupillary anomalies [2, 7]. In Houlden et al.' report of the neurological phenotype of genetically confirmed TAS, CN involvement was present in all patients within a variable range of severity. They signify the possibility for bilateral CN XII involvement in four patients from three distinct families. Moreover, in one of these four patients, bilateral CN XI and unilateral CN X deficits were also noted. Those findings highlight the possibility that our patient could have presented a subclinical form of involvement that deteriorated in the operative setting. Allgrove's syndrome in this patient may also have increased his RLN vulnerability to standard surgical manipulations and inflammatory changes in the early postoperative setting, leading to the course of early deterioration after successful extubation.

In this regard, medical literature is ripe with case reports and series of lower cranial nerve (CN) palsies occurring following uncomplicated anesthesia, both with endotracheal intubation (orotracheally or nasotracheally) and with supraglottic devices such as laryngeal mask airway. Unilateral or bilateral RLN involvement is reported. Evolution is usually benign, and partial or complete recuperation is expected within 6 months of onset.

This case report faces limitations in the absence of a clear etiology to the subject's BRLNP. It cannot be excluded that this complication was caused solely by delayed-onset neurapraxia induced by nerve dissection or by anesthesia and airway management.

The extremely low incidence rate of TAS is a challenge in the proper evaluation of risk factor interactions and of complication occurrences in this population.

## 4. Conclusion

This case report is of a delayed-onset progressive BRLNP after total thyroidectomy for PTC in a patient with triple A syndrome. Postoperative gross anatomical RLN integrity was confirmed with intraoperative nerve monitoring and visual assessment. In the light of this presentation's very low occurrence rate, we suggest that cranial neuropathic involvement due to TAS is at the origin of this complication. This case warrants for increased vigilance for early respiratory distress following successful extubation in TAS patients undergoing anterior compartment neck surgery, as this population might be at increased risk for life-threatening BRLNP. It is not known whether the vulnerability of the RLNs to surgical or anesthetic trauma and subsequent loss of function were a direct consequence of the polyneuropathy. With the increasing interest for outpatient thyroidectomy, adequate patient selection to minimize risk of severe adverse events is paramount. By design and content limitations, this case report cannot formally identify novel risk factors for RLN injury, but may raise clinician awareness on unusual patient characteristics requiring closer postoperative surveillance.

## Data Availability

Data sharing is not applicable to this article as no new data were created or analyzed in this study.
